# The Costs and Cost-Effectiveness of a Two-Dose Oral Cholera Vaccination Campaign: A Case Study in a Refugee Camp Setting in Thailand

**DOI:** 10.3390/vaccines12111235

**Published:** 2024-10-30

**Authors:** Aaron S. Wallace, Kashmira Date, Sarah W. Pallas, Nuttapong Wongjindanon, Christina R. Phares, Taiwo Abimbola

**Affiliations:** 1 Global Immunization Division, US Centers for Disease Control and Prevention, 1600 Clifton Rd NE, Mailstop H24-2, Atlanta, GA 30329, USA; 2 Thailand Ministry of Public Health, U.S. Centers for Disease Control and Prevention Collaboration, Ministry of Public Health, Tiwanon Road, Nonthaburi 11000, Thailand; 3 Division of Global Migration Health, Centers for Disease Control and Prevention, 1600 Clifton Rd NE, Mailstop H16-4, Atlanta, GA 30329, USA

**Keywords:** cholera, oral cholera vaccine, vaccination campaign, cost, cost-effectiveness

## Abstract

Oral cholera vaccination (OCV) campaigns are increasingly used to prevent cholera outbreaks; however, little is known about their cost-effectiveness in refugee camps. We conducted a cost-effectiveness analysis of a pre-emptive OCV campaign in the Maela refugee camp in Thailand, where outbreaks occurred with an annual incidence rate (IR) of up to 10.7 cases per 1000. Data were collected via health sector records and interviews and household interviews. In the base-case scenario comparing the OCV campaign with no campaign, we estimated the campaign effect on the cholera IR and case fatality rate (CFR: 0.09%) from a static cohort model and calculated incremental cost-effectiveness ratios for the outcomes of death, disability-adjusted life-years (DALYs), and cases averted. In sensitivity analyses, we varied the CFR and IR. The household economic cost of illness was USD 21, and the health sector economic cost of illness was USD 51 per case. The OCV campaign economic cost was USD 289,561, 42% attributable to vaccine costs and 58% to service delivery costs. In our base case, the incremental cost was USD 1.9 million per death averted, USD 1745 per case averted, and USD 69,892 per DALY averted. Sensitivity analyses that increased the CFR to 0.35% or the IR to 10.4 cases per 1000 resulted in a cost per DALY of USD 15,666. The low multi-year average CFR and incidence of the cholera outbreaks in the Maela camp were key factors associated with the high cost per DALY averted. However, the sensitivity analyses indicated higher cost-effectiveness in a setting with a higher CFR or cholera incidence, indicating when to consider campaign use to reduce the outbreak risk.

## 1. Introduction

In 2017, there were an estimated 21 million refugees globally, with approximately one-third living in camps and nearly one-half under the age of 14 [1]. Refugee camps can be particularly vulnerable to outbreaks of cholera disease due to crowding, poor sanitation, and poor housing infrastructure [2]. Bacteria that cause cholera infections are generally spread by contaminated water and food. Once contracted, cholera can cause severe diarrhea, leading to dehydration and death [3]. In cholera epidemics within refugee camp settings, case fatality rates (CFRs) can vary between 1% to over 50%, with this wide variation being due to treatment resource availability and sanitation-related behavioral factors to effectively manage these outbreaks [4,5,6]. Overcrowding and precarious conditions, such as those in refugee settings, may create an environment conducive to its rapid spread.

Increasingly, mass cholera vaccination campaigns are used to help reduce the incidence of or eliminate cholera in high-risk or outbreak settings [4]. In a 2017 position statement, the WHO recommended OCV use in endemic settings [3]. Prior experiences with OCV use (mainly Dukoral^®^, manufactured by Valvena Sweden AB, Solna, Sweden) in stable refugee settings have suggested the feasibility and utility of cholera vaccination in these situations, indicating that there may be real gains in cholera prevention and control with OCV use in such settings. As of 2024, the World Health Organization has pre-qualified three oral cholera vaccines: Dukoral^®^, Shanchol™ (manufactured by Sanofi Healthcare India Private Limited, Mumbai, India), and Euvichol-Plus^®^ (manufactured by EuBiologics Co. Ltd., Seoul, Republic of Korea) [3]. Dukoral^®^ is administered with a buffer solution and requires two doses for two years of protection for individuals over 5 years of age and three doses for children aged 2–5 years. Shanchol™ and Euvichol-Plus^®^ do not require a buffer solution and can be safely given to individuals over 1 year of age, with two doses providing 3 years of protection. All three vaccines have a good safety profile based on results from multiple clinical trials [3].

Studies to document the impact, costs, and cost-effectiveness of OCV campaigns have been conducted in Tanzania, Bangladesh, and Indonesia [7,8,9,10]. Additional studies in Ethiopia, Haiti, and Malawi have examined OCV campaign costs only [11,12]; however, there are no published studies on the costs and cost-effectiveness of an OCV campaign within a refugee camp setting, in which the costs and cost-effectiveness may differ. In this study, we estimated the costs of the 2013 OCV campaign, the cost of cholera illness, and the cost-effectiveness of the vaccination campaign in a refugee camp setting [13]. Our findings aim to broadly inform the value of OCV vaccination for specialized populations, such as the Maela refugee camp in Thailand. In particular, the Thailand Ministry of Population and Health viewed the implementation of this OCV campaign as an effort to gain experience and develop strategies for OCV delivery in cholera-prone provinces and populations in the future; thus, conducting a cost-effectiveness analysis was a component of their analytical strategy.

## 2. Method

### 2.1. Study Setting and Population

The Maela camp is home to approximately 50,000 people who fled from Burma during the 1980s after a military regime came into power in Burma; it has been run by the United Nations High Commissioner for Refugees since 1984. The camp is located in a valley with a single road linking the camp to the nearest Thai city of Mae Sot, which is about one hour away by car. In 2005, the non-governmental organization (NGO) Premiere Urgence-Aide Medicale Internationale (PU-AMI) took over healthcare, water, and sanitation services for Maela and two other refugee camps.

The Maela camp has suffered multiple cholera outbreaks over the past 20 years, with the last confirmed cholera case occurring in 2010. In 2013, officials conducted a two-dose oral cholera vaccine (OCV) campaign for all 46,000 refugees in the camp to preemptively protect the population against a cholera outbreak. The 2013 OCV campaign used the Shanchol^TM^ vaccine, which shares similar vaccine characteristics to the Dukoral vaccine in that both do not require a buffer solution for administration, and both can be given to all individuals over one year of age. Additional campaign details are documented elsewhere [13].

Lab-confirmed or presumed cases of cholera occurred in the camp in 2005, 2007, 2008, and 2010 [7]. These four outbreaks lasted between 17 and 26 weeks, with a total of 691 lab-confirmed cases and 1540 suspected cases during the 8-year period (2005–2012) leading up to the OCV campaign. The vast majority of reported cholera cases in Thailand were based in the Maela camp. Before 2008, some patients presenting with acute watery diarrhea (AWD) during lab-confirmed outbreaks were presumptively diagnosed with cholera, whereas after 2008, all patients seeking care for AWD during a confirmed outbreak were tested for cholera. During 2005–2012, the reported annual IR for confirmed or presumed cases ranged between 0.0 and 10.7 cases per 1000 refugees, with an annual average of 3.0 cases per 1000 individuals [13].

### 2.2. Cost Analysis

The cost analysis of the 2013 OCV campaign was conducted from the health sector and household perspectives. Cost data for the campaign were collected via reviews of financial records and interviews with involved organizations, and the costs of cholera illness were collected using household and health worker interviews. We calculated the costs separately by the health sector cost of illness (COI) per case, household COI per case, and OCV campaign cost. All costs were adjusted for inflation from 2013 to 2021 using the Thailand consumer price index and then converted to 2021 USD using the 2021 exchange rate of THB 33.98 to USD 1 [14,15]. The data analysis was conducted using Microsoft Excel 2013.

#### 2.2.1. Health Sector Cost of Illness

We conducted semi-structured interviews of PU-AMI staff to ascertain the time spent treating cases of AWD and abstracted data from PU-AMI budgets of cholera treatment centers (CTCs) that were set up in previous cholera outbreaks. In the Maela camp, a CTC is only set up once a cholera case is confirmed; otherwise, the routine health sector in the camp is used for treating AWD cases and detecting and treating the first few suspected cholera cases in an outbreak. The health sector COI fixed costs comprised items and resources required to set up a cholera care and treatment center, such as tables, medical devices, chairs, water basins, and mats. The health sector COI variable costs included non-reusable items used in cholera care and treatment centers, such as cups, gloves, linens, and plastic bags, as well as variable costs of healthcare provider work hours per treated cholera case. The financial records contained information on CTC set-up expenses, medical supplies, patient material costs, and medicine costs. We calculated the health sector mean variable COI per case across the sample of cases, using the number of confirmed cases that occurred in the last Maela cholera outbreak of 2010 (N = 392).

#### 2.2.2. Household Cost of Illness

As no cases of cholera had occurred in the camp since 2010, we used the cost of AWD illness as a proxy for the household COI for cholera. The illnesses generally have similar effects with regard to the length of illness and likely impact on days of work lost. In 2013, after the campaign, 40 primary cases of AWD were prospectively identified during discharge from health facilities and interviewed using a standardized questionnaire. For the purpose of this evaluation, we defined a case of AWD as any person aged 2 years or older who had three or more loose stools over 24 h, with or without concurrent vomiting. If the case had a caregiver, we interviewed the caregiver to ascertain workdays lost for the caregiver. We collected data on age, gender, monthly income, work details, travel time to/from the health facility, any incurred travel costs, length of diarrheal illness in days, length of time spent at the health facility, related healthcare costs (medicine, food, and treatment service payments), and number of days of missed work. We aimed to enroll 40 cases (based on the expected monthly volume of cases coming to facilities and the planned length of the data collection period) and conducted all interviews within one week of facility discharge. For cases of 2–17 years of age, the caregivers were interviewed about expenses incurred related to the case patient’s illness as younger individuals may be unable to directly recall this information; for case patients of >17 years of age, the case patient was interviewed about their expenses related to the illness. Verbal consent was obtained from all interviewees. We calculated the mean household COI (at one week post-discharge) as the sum of the mean direct cost (i.e., the medical and non-medical costs incurred by households to treat or prevent illness) and the mean indirect cost (i.e., the monetary value of average daily wages lost by case patients and caregivers during the illness period) per case.

#### 2.2.3. OCV Campaign Costs

PU-AMI, the Thailand Ministry of Public Health (MOPH), and the US Centers for Disease Control and Prevention (CDC) sponsored and implemented the two-dose OCV campaign in 2013. Two eight-day vaccination rounds occurred for all eligible individuals (over 1 year of age and not pregnant), with a two-day third round for those who had only received one OCV dose. The rounds were two weeks apart. In the two main vaccination rounds, 43,485 individuals were targeted. The first dose coverage was 81%, and the second dose coverage was 64%. Of the 90,000 OCV doses ordered, 63,085 OCV doses were administered, and 993 doses were wasted [13]. The 25,950 unused doses were repurposed for other OCV campaigns in Thailand, so only the 64,078 used doses were incorporated into the campaign cost estimates. The involved organizations provided cost data for the vaccine and service delivery from the health sector perspective. Detailed information about the campaign preparation and implementation is published elsewhere [13]. In short, PU-AMI conducted community-level informational meetings, circulated newsletters leading up to the campaign, and then set up vaccination campaign posts throughout the community during the campaign. Each post was staffed by 20–25 workers who screened individuals for eligibility (over 1 year of age and not pregnant) and issued vaccination cards. Costs from the household perspective to receive vaccination (i.e., costs and time spent on transport to and from the vaccination site) were calculated based on information provided through the survey of the 40 AWD cases regarding the duration of time traveling to/from a health facility, the proportion of those cases that reported paying for transport to/from the health facility, the proportion of cases who reported working, and their reported monthly salary.

We calculated the direct, indirect, and total OCV campaign costs and the cost per individual targeted and per fully vaccinated individual (FVI) from a societal (health sector and household) perspective. Direct OCV campaign costs included household costs of transport to the campaign session site, and indirect OCV campaign costs encompassed productivity losses due to time spent traveling to/from and receiving the vaccination at the vaccination post.

#### 2.2.4. Cost-Effectiveness Analysis

To calculate the incremental cost-effectiveness, we compared a scenario where an OCV campaign occurred with a scenario where no OCV campaign occurred. In each scenario, we calculated the cost of treatment, OCV campaign, number of estimated cases, deaths, and DALYs and then calculated the differences between each scenario to arrive at an incremental value for each output (Table 1). Our analytic horizon was the three-year period after the campaign occurred, based on the estimated length of protection after OCV vaccination [3].

### 2.3. Incremental Effectiveness of OCV Campaign

We calculated the incremental effect of the campaign by calculating the difference in disease outcomes (deaths, cases, and DALYs) between each scenario (campaign versus no campaign) using a static cohort model. We used 2005–2013 data on the annual cholera incidence rate (IR) and annual cholera deaths derived from the disease surveillance system managed by PU-AMI and MOPH in the camp for the non-campaign scenario.

We then estimated the effect of the OCV campaign on cholera incidence, incorporating both the surveillance system data and other information on life expectancy after infection, disability weights, and duration of immunity as outlined in the approaches by Jeuland and Troeger [8,9] that are based on approaches outlined in the Disease Control Priorities Project [17].

The majority of data used in the scenario calculations were derived from our primary data collection efforts in the Maela camp and from the PU-AMI-managed disease surveillance system data, including the 2005–2013 observed CFR of 0.09%. The external data used were for vaccine efficacy (65% for two doses), disability weight for cholera (0.202), length of OCV protection (three years), and life expectancy at the age of infection (51 years) [8,9,18,19,20] (Table 1).

### 2.4. Incremental Net Cost of OCV Campaign Versus No OCV Campaign

We calculated the incremental cost of the campaign by calculating the difference in cost between each scenario (campaign versus no campaign). We used the following formula to calculate this difference:

Incremental cost of OCV campaign = [(Household COI + health sector COI) × (number of cases with OCV campaign) + (OCV campaign cost)] − [(Household COI + health sector COI) × (number of cases without OCV campaign)]

### 2.5. Incremental Cost-Effectiveness Ratio Calculation

We calculated the incremental cost-effectiveness ratios (ICER) for deaths, cases, and DALYs averted by the campaign by dividing the incremental cost of the campaign by the incremental effects of the campaign over the three-year period of assumed constant vaccine protection of 65%. To calculate the difference in DALYs between the campaign and non-campaign scenario (i.e., DALYs averted), we used the following formulas, assuming no age weighting:

Years of life lost to disability (YLD) averted_t_ = [((1 − CFR) × (vaccine efficacy_i_) × (OCV campaign coverage) × (Population size) × (disease incidence)) × (duration of illness) × (disability weight)]

Years of life lost (YLL) averted = [(CFR × (vaccine efficacy) × (population size) × (OCV campaign coverage) × (disease incidence))/0.03] × [1 − exp(−0.03 × (life expectancy)]

DALYs averted_t_ = YLD_t_ averted + YLL_t_ averted

$$Total\;DALYs\;averted=\sum_{t=0}^{2}(DALYs\;{averted}_{t})/{\left(1+0.03\right)}^{t}$$

where *t* is the year of the estimate (t = 0 is the first year after the vaccination campaign, and t = 2 is the third year after the vaccination campaign), and CFR is the observed case fatality rate for 2005–2013 (0.09%). We also conducted sensitivity analyses by varying the IR, CFR, campaign coverage, and COI to determine how much these indicators influenced the cost-effectiveness of the intervention. Lastly, we used the current guidance provided by the Thailand Health Intervention and Technology Assessment Program (HITAP) within the Ministry of Health to assess the health intervention cost-effectiveness as a proxy reference for a cost-effectiveness threshold for our analysis [21,22]. Based on research completed in the Thai healthcare setting on willingness to pay for a quality-adjusted life year (QALY), the current cost-effectiveness threshold, set in 2013, is THB 160,000, equivalent to USD 4708 in 2021 USD. This latter threshold was considered a proxy in this study, as we calculated an ICER in cost per DALYs. Previous studies have indicated the relevance of using QALYs and DALYs interchangeably [23].

## 3. Results

### 3.1. Household and Health Sector Cost of Illness

From the household perspective, the average direct and indirect COI per case were USD 3 and USD 18, respectively, summing up to a household economic COI of USD 21 per case or 30% of the mean monthly household income (USD 72) among surveyed households (Table 2). The direct medical costs were USD 1 per case, and the direct non-medical costs were USD 2, including transport costs (USD 0.20 per case) and food costs (USD 2). The indirect costs encompassed productivity losses due to time spent on treatment at the facility (USD 5 per case), time spent on travel (USD 2 per case), and time spent with illness at home (USD 13 per case). At the health sector level, the fixed and variable COI per case were USD 26 (52%) and USD 25 (48%), respectively (Table 2), summing up to a health sector economic COI of USD 51. The combined household and health sector economic COI was USD 72 per case, of which 72% was health sector costs, while 28% was household costs.

### 3.2. OCV Campaign Cost

The total cost of the OCV campaign was USD 289,561, consisting of USD 239,537 (80%) in health sector costs and USD 57,589 (20%) in household costs (Table 3). The total campaign costs per fully vaccinated individual (FVI) and per targeted individual were USD 10.47 and USD 6.67, respectively. The health sector direct costs included USD 122,977 for the vaccine used in this campaign (42% of total campaign costs, with USD 4.45 per FVI). The purchase price for two doses of the vaccine (a full course for a single individual) was USD 3.82. For vaccination delivery activities in the two campaign rounds, the health sector cost was USD 108,993 (38% of the total campaign costs, with USD 3.94 per FVI and USD 2.51 per targeted individual). From the health sector perspective, the vaccination delivery direct costs included USD 48,910 for cold chain equipment and fuel, USD 24,488 for vaccination materials (health cards, cups, and water), USD 14,985 for campaign staff remuneration, USD 13,984 for community mobilization materials, and USD 7115 for the transport of vaccines and materials. From the household perspective, the vaccine delivery direct costs consisted of USD 12,611 for transport costs (USD 0.46 per FVI), and the indirect costs consisted of USD 44,978 (USD 0.81 per FVI) in lost wages due to the household time spent attending the campaign. Based on a total of 63,057 doses administered (including first and second doses), the total cost per dose delivered was USD 4.59, the health sector cost per dose delivered was USD 3.68, and the household cost per dose delivered was USD 0.91.

### 3.3. Cost-Effectiveness

In the base case scenario, the OCV campaign was estimated to have averted 161 cases, 0.15 deaths, and 4.03 DALYs over the course of the 3-year duration of vaccine protection at an incremental cost of USD 281,336 (Table 4). The ICER for each outcome was USD 1,939,424 per death averted, USD 1745 per case averted, and USD 69,892 per DALY averted.

In the sensitivity analysis scenarios, the ICER per DALY averted decreased to USD 15,666, with an annual incidence rate of 10.4 cases per 1000 individuals, and to USD 15,603 or a CFR of 0.35%, roughly three times the Thai cost-effectiveness threshold. If the OCV campaign had achieved 100% two-dose coverage, the ICER per DALY averted would have decreased to USD 57,621. If both the household and health sector COI were to be included at USD 72 per case, the ICER per DALY averted would decrease to USD 56,638.

If we assume the campaign to be the reason for the lack of any reported cholera cases during the three-year time horizon after the campaign, the cost per death and DALY averted would decrease to USD 559,416 and USD 52,811, respectively.

## 4. Discussion

In our study, the first to examine the cost-effectiveness of an OCV campaign in a refugee camp setting, we estimated that the incremental cost of the OCV campaign was USD 281,336 in our base case scenario, while the ICERs per death averted, per DALY averted, and per cholera case averted were USD 1.9 million, USD 69,892 and USD 1745, respectively. The aim of our study was not to inform a particular government decision in Thailand, and our findings suggest that this campaign would not be cost-effective using the willingness to pay thresholds (WTPs), such as the World Health Organization’s three times gross domestic product per capita or the Health Intervention and Technology Assessment Program (HITAP) [24]. There is no conventional WTP threshold recommendation for refugee settings; however, it is noteworthy that the campaign could be cost-effective using the WHO thresholds in the scenario of higher cholera incidence rates (>10.0 cases per 1000 individuals) or a higher cholera CFR (>0.35%). The historical average annual CFR in the Maela camp is extremely low compared with other country settings where cholera outbreaks have occurred, with CFRs reported between 0.5% and 3% [7,8,9,10]. The very low CFR in Maela might indicate the ability of the local NGO, PU-AMI, to respond quickly with treatment for cholera cases when outbreaks have occurred, thereby nearly eliminating the risk of cholera-related death over the past ten years. Considering the vulnerability of refugee camps to outbreaks of cholera disease due to crowding, poor sanitation, and poor housing infrastructure, global health decision-makers, including non-governmental organizations financially responsible for refugee health, could consider the value of cholera vaccination in settings of high cholera incidence and high CFRs.

We used a static cohort model to estimate the post-campaign effects on cholera incidence and death, with 65% efficacy with two doses of the Shankol^TM^ vaccine, and, thus, the continued occurrence of cholera cases; however, surveillance system data from 2012 to 2015 indicate no cholera cases detected since the campaign.

In recent studies from Tanzania and elsewhere [7,8,9], results about the cost-effectiveness of OCV campaigns are mixed, largely due to variations in the assumed disease incidence rates and CFRs or the GDP per capita upon which the thresholds for cost-effectiveness are based. Higher health sector COI values were observed in Maela compared with Indonesia, India, and Bangladesh but lower compared with Tanzania. The household COI in Maela was about half the reported household COI from Tanzania. Costs for vaccine administration and the vaccine (USD 6.67 per targeted individual) were well below those reported elsewhere, which may be largely due to the confined nature of the camp and the decreasing cost of the Shankol^TM^ vaccine. Due to the relatively lower costs of OCV in the Maela camp campaign, the cost ratio between vaccine and delivery was about 50:50; in comparison, the Tanzania study, as well as studies from Vietnam, reported a ratio of approximately 25:75.

A number of study limitations exist. We used a convenience sample of household members, which may not represent the costs of illness for all cases. These individuals only provided information about costs one week after illness, so costs of any long-term sequelae (e.g., coma or renal failure) were not included. We also used the cost of AWD illness as a substitute for the cost of cholera illness. To the extent that cholera-specific symptoms and sequelae may differ from those of other causes of AWD, our COI estimates may not accurately reflect the cholera-specific COI [9,10]. The data we used to estimate the effects of the OCV campaign were based on historical surveillance system data, which could underestimate the true number of cholera cases that occurred [23]; however, due to the small and confined geographical nature of the camp and the active engagement of PU-AMI in the area, the surveillance system is likely more sensitive than similar systems in other low- and middle-income countries, where data quality can be a concern [25]. These results can remain valuable for many years into the future; however, it would be important to make additional inflation-associated adjustments to compare with the current costs of care. Shanchol^TM^, alongside Euvichol^TM^, which is essentially the same vaccine, are the main vaccines used for mass vaccination campaigns; thus, this analysis of a Shanchol^TM^-based 2013 campaign continues to remain relevant for decision-makers.

## 5. Conclusions

Our sensitivity analysis indicates the key drivers under which this campaign would be substantially more cost-effective and should be key considerations when deciding on the use of an OCV campaign in a refugee camp setting. The cholera incidence rates and CFRs at which the OCV campaign would have been substantially more cost-effective were observed in Maela in one previous year of our study time frame and were at levels that are likely to be observed in other refugee camp settings where the infrastructure and organizational management may be much weaker than those available in the Maela camp. Our findings provide evidence to support the continued consideration of OCV campaigns in settings likely to have high incidence and case fatality rates as a key strategy for reducing the risk of cholera outbreaks.

## Figures and Tables

**Table 1:** External inputs for burden of cholera disease and oral cholera vaccine (OCV) campaign effectiveness conducted in Maela camp, Thailand, in 2013 for calculating cost-effectiveness.

Component	Description	Data Source	Base Case Value (Sensitivity Analysis Value)
Observed average cholera incidence	Historical cholera incidence observed in camp (2005–2013)	Government disease surveillance system	3.0 (10.0)
Cholera case fatality rate	Historical cholera case fatality rate observed in camp (2005–2013)	Government disease surveillance system	0.09% (0.35%)
Cholera illness duration	Average duration of cholera illness in days	[9]	4 days
Life expectancy	Average life expectancy	Government surveillance system data and World Bank [14]	51 years
OCV effectiveness	Vaccine effectiveness for two doses of Shanchol^TM^ vaccine	[9]	65%
OCV duration of protection	Duration of vaccination protection for Shanchol^TM^ vaccine	[9]	3 years
OCV campaign coverage	2013 OCV campaign 2-dose coverage	[13]	64% (100%)
GDP per capita	2017 Thailand GDP per capita	[14]	USD 6593
DALY weight	DALY weight for cholera illness	[16]	0.202

COI: cost of illness; OCV: oral cholera vaccination; VE: vaccine effectiveness; GDP: gross domestic product; DALY: disability-adjusted life years. Monetary values given in 2017 USD unless otherwise stated.

**Table 2:** Direct and indirect costs per case of cholera illness by perspective (household or health sector) in Maela camp in Thailand in 2013 in 2021 USD.

Household Cost of Illness	Mean Cost per Case (Standard Deviation)	% Total Cost
Direct medical		
Medicine and treatment services	USD 1.19 (USD 0.31)	6%
Direct non-medical		
Food	USD 1.85 (USD 0.44)	9%
Transport	USD 0.20 (USD 0.03)	1%
Indirect		
Lost income for case	USD 6.76 (USD 1.42)	32%
Lost income for caregiver	USD 11.03 (USD 2.36)	53%
Total household COI	USD 21.03 (USD 3.91)	100%
Health sector cost of illness		
Fixed cost	USD 26.43 (USD 4.14)	52%
Variable cost	USD 24.60 (USD 4.01)	48%
Total health sector COI	USD 51.03 (USD 7.93)	100%
Total COI (household + health sector)	USD 72.06 (USD 9.48)	

**Table 3:** Total costs per targeted and fully vaccinated individual (FVI) associated with 2013 oral cholera vaccine (OCV) campaign in Maela camp in Thailand in 2021 USD.

	Total ^1^	Per Targeted Individual ^2^	Per FVI ^3^	Per Dose
Health sector cost				
Vaccine	USD 122,977 ^1^	USD 2.83	USD 4.45	USD 1.95
Service delivery	USD 108,993	USD 2.51	USD 3.94	USD 1.73
Household direct cost				
Transport	USD 12,611	USD 0.29	USD 0.46	USD 0.21
Household indirect cost				
Lost wages	USD 44,978	USD 1.04	USD 0.81	USD 0.70
Total cost	USD 289,561	USD 6.67	USD 10.47	USD 4.59

^1^. Total costs for two OCV rounds with target population of 43,485 individuals per round, with 81% coverage in first round and 64% coverage in second round. ^2^. Cost per targeted individual based on target population of 43,485 individuals. ^3^. Cost per fully vaccinated individual (FVI), where 64% of targets received two OCV doses (fully vaccinated).

**Table 4:** Cost-effectiveness of 2013 oral cholera vaccine (OCV) campaign in base case scenario in Maela camp in Thailand in 2021 USD.

	Vaccination	No Vaccination	Difference (Vaccination Minus No Vaccination)
Effects			
Number of cases	226	387	−161
Number of deaths	0.20	0.35	−0.15
DALY averted over 3-year period of vaccination protection ^1^	5.77	9.80	−4.03
Costs, 2021 USD			
Cost of vaccination program	USD 289,561	USD 0	USD 289,561
Health sector cost of illness	USD 11,547	USD 19,772	−USD 8225
Cost of treatment and vaccination program (incremental cost)	USD 301,108	USD 19,772	−USD 281,336
Cost-effectiveness ratios, 2021 USD			
ICER (case): incremental costs/case averted			USD 1745
ICER (death): incremental costs/death averted			USD 1,939,424
ICER (DALY): incremental costs/DALY (over duration of vaccination protection ^1^) averted			USD 69,892

^1^: duration of vaccine protection against cholera estimated at 3 years, using a 3% discount rate.

## Data Availability

The datasets used and/or analyzed during the current study are available from the corresponding author upon reasonable request.
